# Recurrent Inflammatory Myositis as an Extra-Intestinal Manifestation of Dormant Ulcerative Colitis in a Patient on Long-Term Mesalamine

**DOI:** 10.1155/2019/2090379

**Published:** 2019-04-07

**Authors:** Srikanth Naramala, Venu Madhav Konala, Sreedhar Adapa, Pravallika Amudala, Romeo C. Castillo, Nikhil Agrawal, Hemant Dhingra

**Affiliations:** ^1^Department of Rheumatology, Adventist Medical Center, Hanford, CA, USA; ^2^Ashland Bellefonte Cancer Center, Ashland, KY, USA; ^3^The Nephrology Group, Fresno, CA, USA; ^4^Family Practice, Kakatiya Medical College, Warangal, Telangana, India; ^5^Hanford Family Medicine Residency, Hanford, CA, USA; ^6^Nephrology, Beth Israel Deaconess Medical Center, Harvard Medical School, Boston, MA, USA; ^7^Internal Medicine Residency, St Agnes Medical Center, Fresno, CA, USA

## Abstract

Ulcerative colitis (UC) is a chronic systemic inflammatory condition primarily involving the large bowel mucosa with relapsing and remitting episodes. It is also associated with multiple extra-intestinal manifestations [EIM], including skeletal muscle involvement which is rare. Review of the literature reported only a few cases of inflammatory myositis in association with UC. We report an unusual presentation of recurrent inflammatory myositis of lower extremities in a 28-year-old male with quiescent UC and on long-term mesalamine therapy.

## 1. Introduction

Ulcerative colitis is a chronic systemic large bowel inflammatory condition with an incidence of 2.2 to 14.3 cases per 100,000 person-years [1]. It is more common among ex-smokers and nonsmokers [1]. EIM accounts for 25% of patients with inflammatory bowel disease in their lifetime [2, 3]. Review of the literature reported only a few cases of inflammatory myositis in association with UC. The onset of myositis can present anytime during the course of the disease, but more common with acute flare-ups. We report an unusual presentation of recurrent inflammatory myositis of lower extremities in a patient with quiescent UC and on long-term mesalamine therapy.

## 2. Case Report

A 28-year-old Hispanic male painter with no past medical history presented to the emergency department with progressively worsening bloody diarrhea and diffuse myalgias mainly localized to bilateral lower extremities for the last three weeks. He denied any recent travel, sick contacts, or taking any new medications including antibiotics. He denies any change in his diet. He was not on any medications including over-the-counter medication at the time of admission. The patient reported throat discomfort few days prior to admission and was presumptively diagnosed with Streptococcal sore throat by his primary care physician. He received a 10-day course of amoxicillin without benefit.

His admission complete blood count (CBC) revealed a white cell count of 17,600 mm^3^ and hemoglobin of 9.7 g/dl, decreased to 8.9 g/dl over the next 3 days. Other laboratory values revealed a sedimentation rate of 114 mm/hr and CRP 33.59 mg/dL. Liver function tests showed elevated alkaline phosphatase at 183 IU/l and AST 145 IU/l. CPK was elevated 1433 IU/l. Basic metabolic panel was unremarkable except potassium low at 2.7 mmol/l. Hepatitis panel, HIV serology, serum coccidioidomycosis, urine gonorrhea, and chlamydia PCR were all negative.

Stool culture was negative for Escherichia coli, Salmonella, Shigella, parasites, and Clostridium difficile by PCR. Blood cultures were negative.

Patient had an echocardiogram during the hospitalization which was reported as normal. He had left lower extremity swelling and US venous Doppler of left lower extremity was negative for deep vein thrombosis. He had a CT Abdomen and pelvis with contrast which was normal.

Gastroenterologist was consulted, who did colonoscopy, and the patient was found to have diffuse colitis from cecum all the way to the rectum, without skip lesions. Terminal ileum was normal. Biopsies of the colon showed marked crypt architectural irregularity with multiple crypt abscess. The lamina propria showed mixed inflammatory cells with prominence of plasma cells, neutrophils, and lymphoid aggregates with histologic features consistent with ulcerative colitis. Granulomas were not identified.

During the course of his hospitalization, he developed fever with temperature of 101.8F. He was started on IV fluids with 0.9% sodium chloride, mesalamine, and prednisone taper which dramatically improved his symptoms. His elevated Creatine phosphokinase (CPK) levels were thought to be secondary to rhabdomyolysis from hypokalemia. He was discharged to be followed by primary care physician and his disease was in remission for one year.

**Figure 1:**
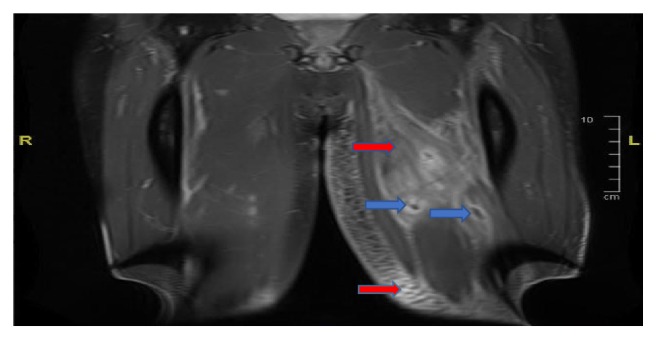
MRI postcoronal T1 of bilateral thighs. Red arrows show extensive subcutaneous edema with enhancement throughout the subcutaneous adipose space of the left thigh as well as extensive infiltration of muscles in multiple compartments with blue arrow showing abscess.

**Figure 2:**
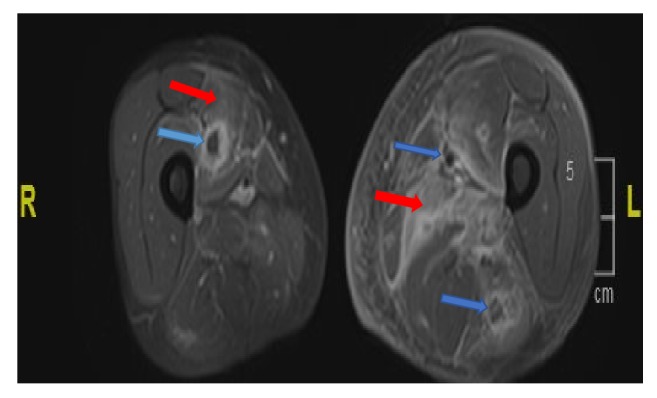
MRI postaxial T1 of bilateral thighs. Red arrows show inflammation in vastus medialis of the right thigh extending up to the periosteum with abscess (Blue arrow). Inflammation and edema of vastus muscle group on the left (Red arrow) with fluid collections in different muscles with abscess (Blue arrow).

He presents to emergency department one year later with worsening bilateral lower extremity pain and difficulty ambulating. He denies any flare up of his UC and is on mesalamine. His CPK was elevated at 9455 IU/L, with abnormal liver function. Patient was febrile with white count of 25,300 mm^3^. He was hydrated aggressively with 0.9% sodium chloride. He continued to have increasing pain localized to the left thigh, left calf, and the inner right thigh. MRI of lower extremities with and without contrast showed numerous intramuscular abscesses in the left thigh, extensive infiltration of the muscles in all compartments of the left thigh with subcutaneous edema, and fewer intramuscular abscesses in the right thigh as shown in Figures 1 and 2. No periosteal reaction was identified. Infectious disease specialist was consulted and he was started on empiric antibiotics. Prednisone was added after initial cultures which were negative. His antibiotics were discontinued. His symptoms improved with above management and was discharged on prednisone taper.

**Figure 3:**
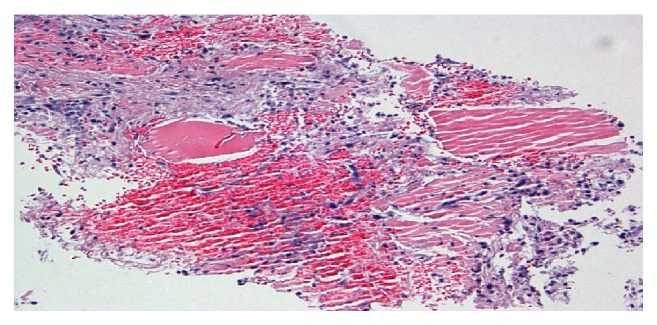
Light microscopy: high power, lysis of myocytes with marked inflammation.

**Figure 4:**
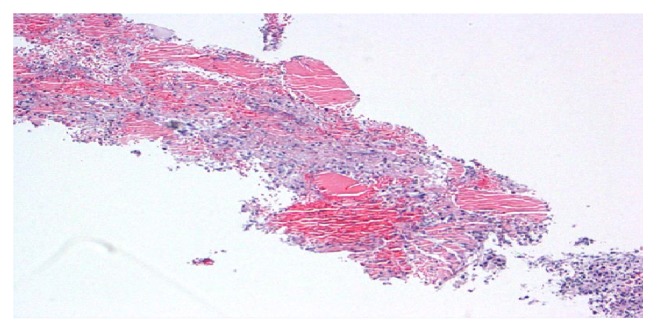
Light microscopy: low power, muscle cell lysis with necrosis.

Left lower leg muscle biopsy showed fragments of myonecrosis with an acute inflammatory exudate as shown in Figures 3 and 4. Acid-Fast Bacillus smear, gram stain, and pan cultures were negative. Immunohistochemical staining and metabolic assays on muscle biopsy were normal. Extensive autoimmune serologies including myositis antibody panel were negative except positive ANA of 1:80.

He had two more hospitalizations with similar presentation involving lower extremities in the next 5 months managed with tapering prednisone course. He has repeat MRIs of his lower extremities with repeat muscle biopsy consistent with similar findings. His UC was under control with mesalamine during all these episodes. He was diagnosed with inflammatory myositis from his underlying dormant UC. Methotrexate was added to mesalamine along with prednisone taper. His prednisone was stopped in 2 months. His symptoms resolved and his mesalamine was tapered off in 8 months. He remained in remission both from myositis and UC for last 12 months on methotrexate 20mg per week, tolerating well without any side effects.

## 3. Discussion

UC is a chronic systemic inflammatory condition primarily involving the mucosa of large bowel associated with relapsing and remitting episodes. It is also associated with multiple extra-intestinal manifestations with prevalence of approximately 25% during the course of the disease as shown in Table 1 [2, 3]. Skeletal muscle involvement is one of the rare manifestations [6, 7].

Review of the literature shows only a few cases describing an association of ulcerative colitis and inflammatory myositis, most of them during acute exacerbations of the disease [6, 8]. In contrast, we report a case of recurrent inflammatory myositis in a patient who was in remission from UC on long-term immunosuppression with mesalamine. Diagnosis of inflammatory myositis should be considered in inflammatory bowel disease patients complaining of myalgia or muscular weakness who are also in remission. The occurrence of EIM can considerably affect morbidity in these patients. Accurately diagnosing and treating these EIM is crucial to improve quality of life in these patients [9].

Myopathy can either be a direct consequence of disease activity of the bowel (e.g., rhabdomyolysis, electrolyte imbalance, and dehydration) or be an immune-mediated response as in our patient. Immune-mediated myositis should be strongly considered as an important differential diagnosis for myopathy in patients with remission [10].

Understanding the pathogenesis of immune-mediated EIM can help with early recognition and adequate and aggressive management, which will contribute to complete recovery and faster remission. In our case, complete remission was achieved with addition of methotrexate to mesalamine, and prednisone taper over two months.

Data on therapy of EIM in IBD patients is scarce and mainly consists of case reports and case series. The advent of biologic drugs has significantly changed the management of EIM in IBD patients [11]. The optimal understanding of immunosuppressant agents, exposure to complex patients with EIM, and exposure to patients with prior therapy failure can help in decision making for escalating therapy for the induction and/or maintenance of remission.

An escalating treatment strategy should be mainly considered in patients with severe disease activity, prior therapy failure, and high relapse risk. An appropriate immunosuppressive therapy should be considered based on patient demographics, disease features, current disease status, and patient's social and economic preferences.

## Figures and Tables

**Table 1:** Extra intestinal manifestations of inflammatory bowel disease [2, 4, 5].

Common	Less Common
(1) Musculoskeletal: Osteoporosis, ankylosing spondylitis, isolated joint involvement such as sacroiliitis (2) Hepatobiliary: Primary sclerosing cholangitis and cholangiocarcinoma, cholelithiasis (3) Ocular: Uveitis iritis, episcleritis, scleromalacia, retinal vascular disease, keratitis (4) Skin and mouth: Reactive lesions - Erythema nodosum, pyoderma gangrenosum, aphthous ulcers, vesiculopustular eruption	(1) Airway and parenchymal lung disease: Pulmonary fibrosis, bronchitis, interstitial lung disease, necrobiotic nodules, laryngotracheitis (2) Cardiac: Pericarditis, myocarditis, endocarditis, heart block, cardiomyopathy (3) Blood and vascular: Thrombocytopenic purpura; thrombophlebitis and thromboembolism, arteritis and arterial occlusion, cutaneous vasculitis (4) Renal and genitourinary tract: Renal tubular damage, amyloidosis, drug-related nephrotoxicity (5) Neurologic: Peripheral neuropathy, myelopathy, vestibular dysfunction, myasthenia gravis. (6) Miscellaneous: Colitis-associated carcinoma, Polymyositis
